# COVID-19 and Respiratory System Disorders: Current Knowledge, Future Clinical and Translational Research Questions

**DOI:** 10.1161/ATVBAHA.120.314515

**Published:** 2020-09-22

**Authors:** Shari B. Brosnahan, Annemijn H. Jonkman, Matthias C. Kugler, John S. Munger, David A. Kaufman

**Affiliations:** 1 Division of Pulmonary, Critical Care, and Sleep Medicine, Department of Medicine, NYU School of Medicine (S.B.B., M.C.K., J.S.M., D.A.K.).; 2 Keenan Centre for Biomedical Research, Critical Care Department, St. Michael’s Hospital, Toronto, Canada (A.H.J.).; 3 Department of Intensive Care Medicine, Amsterdam UMC, location VUmc, Amsterdam, the Netherlands (A.H.J.).

**Keywords:** acute respiratory distress syndrome, COVID-19, diaphragm, infection, respiratory system, thromboembolism

## Abstract

The severe acute respiratory syndrome coronavirus-2 emerged as a serious human pathogen in late 2019, causing the disease coronavirus disease 2019 (COVID-19). The most common clinical presentation of severe COVID-19 is acute respiratory failure consistent with the acute respiratory distress syndrome. Airway, lung parenchymal, pulmonary vascular, and respiratory neuromuscular disorders all feature in COVID-19. This article reviews what is known about the effects of severe acute respiratory syndrome coronavirus-2 infection on different parts of the respiratory system, clues to understanding the underlying biology of respiratory disease, and highlights current and future translation and clinical research questions.

**Graphic Abstract:**

A graphic abstract is available for this article.

**Highlights**

- Severe acute respiratory syndrome coronavirus-2 is a novel coronavirus that emerged in late 2019, causing a pandemic.
- Severe acute respiratory syndrome coronavirus-2 appears to cause predominantly respiratory disease in the form of viral pneumonia.
- Severe cases of severe acute respiratory syndrome coronavirus-2 infection can lead to hypoxemic respiratory failure with features of the acute respiratory distress syndrome.
- Severe acute respiratory syndrome coronavirus-2 infection also may increase the risk of arterial or venous thromboembolism, by mechanisms that are not fully understood.

Coronaviruses are important human pathogens, and research into their behavior is nearly a century old.^1^ In late 2019, a novel coronavirus emerged in Wuhan, China, and then spread worldwide. In February 2020, the World Health Organization designated coronavirus disease 2019 (COVID-19) as the name of the human disease caused by severe acute respiratory syndrome coronavirus-2 (SARS-CoV-2), which was previously known as 2019-nCoV (2019 novel coronavirus).^2^ Viral pneumonia is the most frequent serious clinical manifestation of COVID-19, prominently featuring fever, cough, dyspnea, hypoxemia, and bilateral infiltrates on chest radiography.^3–6^ Dry cough is more common than a productive cough.^6^ Dyspnea appears after a median time of 5 to 8 days.^5,6^ Severe hypoxemic respiratory failure consistent with the Berlin definition of the acute respiratory distress syndrome (ARDS) occurs in a significant proportion of patients with COVID-19 pneumonia.^7,8^ Patients who require mechanical ventilation have a high risk of death.^9^

A detailed understanding of the respiratory pathophysiology of COVID-19 remains elusive, however, because comprehensive data from clinical and research tools such as pulmonary artery catheterization or transpulmonary thermodilution, respiratory mechanics, mixed inert gas elimination technique, or electrical impedance tomography remain lacking or under development.^10–14^ The clinical observations, radiology, and pathology offer clues about the respiratory pathogenesis of COVID-19 and raise interesting research questions. This review will highlight the current knowledge on COVID-19 pathophysiology, with a focus on the effects of SARS-CoV-2 on airway and alveolar epithelium, vascular endothelium, and the control of breathing. We present questions that arise from these observations and indicate possible future research directions that will enhance our understanding of COVID-19 clinical pathophysiology and guide the development of future treatments.

## Clinical, Radiographic, and Pathological Considerations

Clinical findings include hypoxemia, which often appears out of proportion to the sense of dyspnea patients experience. Gattinoni et al^15^ suggest that shunt physiology (perfusion of unventilated respiratory units) may be accompanied by severe abnormalities in ventilation-perfusion (V/Q) matching, with disordered hypoxic vasoconstriction playing a key role. These investigators have also remarked on discrepancies between the severity of hypoxemia and relatively preserved respiratory system compliance, also suggesting that severely abnormal V/Q matching is a prominent feature in ARDS associated with COVID-19. To delineate the contribution of viral infection, immune-mediated damage, or ventilator-associated lung injury to the observed pathophysiology^16^ studies of larger patient populations with well-described associations between the disease course, clinical and radiographic findings, treatments, coinfections, and histopathology will be required.

Radiographic studies of the respiratory system of patients with COVID-19 variably reveal normal lung parenchyma, ground-glass opacities, focal consolidations, and abnormalities of pulmonary vascular perfusion.^17^ Ground-glass opacities in bilateral, peripheral, and lower lobe distribution appear to be the most common pattern on computed tomography (CT) scanning, although systematic reviews suggest that there is no pathognomonic CT pattern.^18–20^ Comparison of CT scans of COVID-19–associated pneumonia to CT scans of other viral pneumonias suggests that the peripheral distribution of opacities, a ground-glass appearance, fine reticular appearance, and vascular thickening are more prominent in COVID-19.^21^

Pathology studies have established insights into the lung pathology caused by SARS-CoV-2 infection. Autopsy studies of patients with COVID-19 have found congested lungs with a patchy distribution of abnormalities on gross examination. Microscopic findings included diffuse alveolar damage (DAD) with hyaline membrane formation, pneumocyte activation, microvascular thrombi, lymphocytic inflammation, and proteinaceous edema.^22,23^ Other autopsy series report vascular remodeling via intussusceptive angiogenesis in the presence of microvascular thrombi.^23^ Other reports note that lung histopathologic findings in COVID-19 are varied and reflect the wide range of abnormalities demonstrated in ARDS from other causes.^24^ A core needle biopsy-based study described areas of fibrosis, chronic inflammation, and loose fibrous plugs associated with organizing pneumonia in addition to ARDS.^25^ A small series of autopsies reported lymphocytic viral pneumonia in patients who died early in the course of the disease and acute fibrinous and organizing pneumonia among patients who died later in the course.^26^ These authors also reported endothelial injury with vacuolization of the cytoplasm and detachment of cells in small and medium-sized pulmonary arteries. Another case series reported deposition of fibrin and erythrocytes in the alveolar spaces and septa as well as hemorrhage and hemosiderin deposition accompanied by complement complex deposition, especially near the alveolar capillaries.^27^ Alveolar type II (AT2) cell hyperplasia, fibrin exudates, vascular congestion, and mononuclear and multinucleated giant cell alveolar inflammation (with a noted absence of neutrophilic inflammation) were reported in 2 patients who underwent resection of lung neoplasms and were found later to have COVID-19.^28^

## Airway Epithelium

The respiratory tract epithelium is the key entry point for beta-coronaviridae, which includes SARS-CoV-2, MERS-CoV (Middle East respiratory syndrome-related coronavirus), and SARS-CoV, into the human host.^29,30^ The airway epithelium acts as a barrier to pathogens and particles, preventing infection and tissue injury by the secretion of mucus and the action of mucociliary clearance while maintaining efficient airflow. Inhaled SARS-CoV-2 particles likely infect different epithelial cell types on their way to the distal lung. Current observations suggest that initial viral contact occurs in the nasal mucosa through binding of the viral S (spike) protein to the ACE2 (angiotensin-converting enzyme-2) receptor, followed by cleavage of S protein by TMPRSS2 (transmembrane serine protease 2). Replication of SARS-CoV-2 within these cells follows.^31–33^ The ACE2 protein is a type I transmembrane metallocarboxypeptidase that converts angiotensin 2 to metabolites, many of which exert vasodilatory properties or interfere with the renin-angiotensin-aldosterone system. Whether manipulating ACE2 levels or activity modifies the risk for developing COVID-19 or ARDS is a potential area of investigation. In vitro data from SARS-CoV indicate that the ciliated airway epithelium serves as a primary site for viral infection; however, whether these airway epithelial cells express sufficient ACE2 to permit viral entry is controversial.^34^

The presence of viral particles in the nasal epithelium is the underlying rationale for obtaining nasopharyngeal material for polymerase chain reaction–based detection of the SARS-CoV-2 genome. Current polymerase chain reaction–based diagnostic tests for SARS-CoV-2 infection lack quantification of viral load and have variable negative and positive predictive values.^35^ Therefore, future research should determine whether using more quantitative assessments of viral RNA or alternative methods of detecting viral genetic material (such as fluorescence-in-situ-hybridization) improve the diagnostic characteristics of SARS-CoV-2 tests, increase the throughput of testing, inform decisions about isolation and contact tracing of individual patients, and assess viability of viral particles. Although nasal epithelial cells appear to demonstrate the highest level of ACE2 expression, the range of cells that express ACE2 is broad.^33^ Understanding qualitative and quantitative patterns of ACE2 or TMPRSS2 expression could provide important clues about which cell types and organs may provide viral portals of entry, identifying targets for interventions aimed at stopping viral entry and replication.

After entering and replicating within the nasal mucosa, SARS-CoV-2 travels to the conducting airways, where it triggers an immune and inflammatory response, manifesting in clinical signs and symptoms of COVID-19.^36^ Infected epithelial cells may express inflammatory mediators such as CXCL10 (C-X-C motif chemokine 10) and interferons.^37,38^ Whether the expression level of these mediators is useful in identifying patients with a higher risk for severe disease is an area of active research. Preliminary reports suggest that small airway ACE2 expression is increased in current smokers and people with COPD (chronic obstructive pulmonary disease), which may partly explain why individuals with underlying cardiopulmonary disease appear to be more likely to die from severe COVID-19.^39,40^ Alternatively, deficiency of ACE2 in people with advanced age, diabetes mellitus, or cardiovascular disease, together with increased clearance of ACE2 from the cell surface with infection, may result in overactivity of the ACE-angiotensin 2-angiotensin 1 receptor axis, leading to increased inflammation and thrombosis.^41^ Observational studies^42^ have not found harm associated with use of ACE inhibitors or angiotensin receptor blockers in patients with COVID-19, and guidelines^43^ recommend continuing such drugs in patients with COVID-19 being treated with them for approved indications. In patients with severe COVID-19 and hypoxemic respiratory failure, thick and copious mucous has been observed, with obstruction of the airways with inspissated mucous reported.^44^ Significant knowledge gaps exist about the behavior of SARS-CoV-2 in infected ciliated epithelial cells of the conducting airways and whether cell damage results in disordered mucociliary function. If this occurs, it could be an explanation for the production and retention of thick mucus (raising airway resistance and obstructing airflow to respiratory units). Whether ACE2 on nasal or lower airway epithelial cells is a viable therapeutic target for preventing viral infection or minimizing viral replication is also a valid research question. Whether treatments such as dornase alfa or N-acetylcysteine, which are directed at lowering the viscosity of airway mucous, are beneficial are reasonable hypotheses to test.

## Alveolar Epithelium and Interstitium

**Figure 1.**
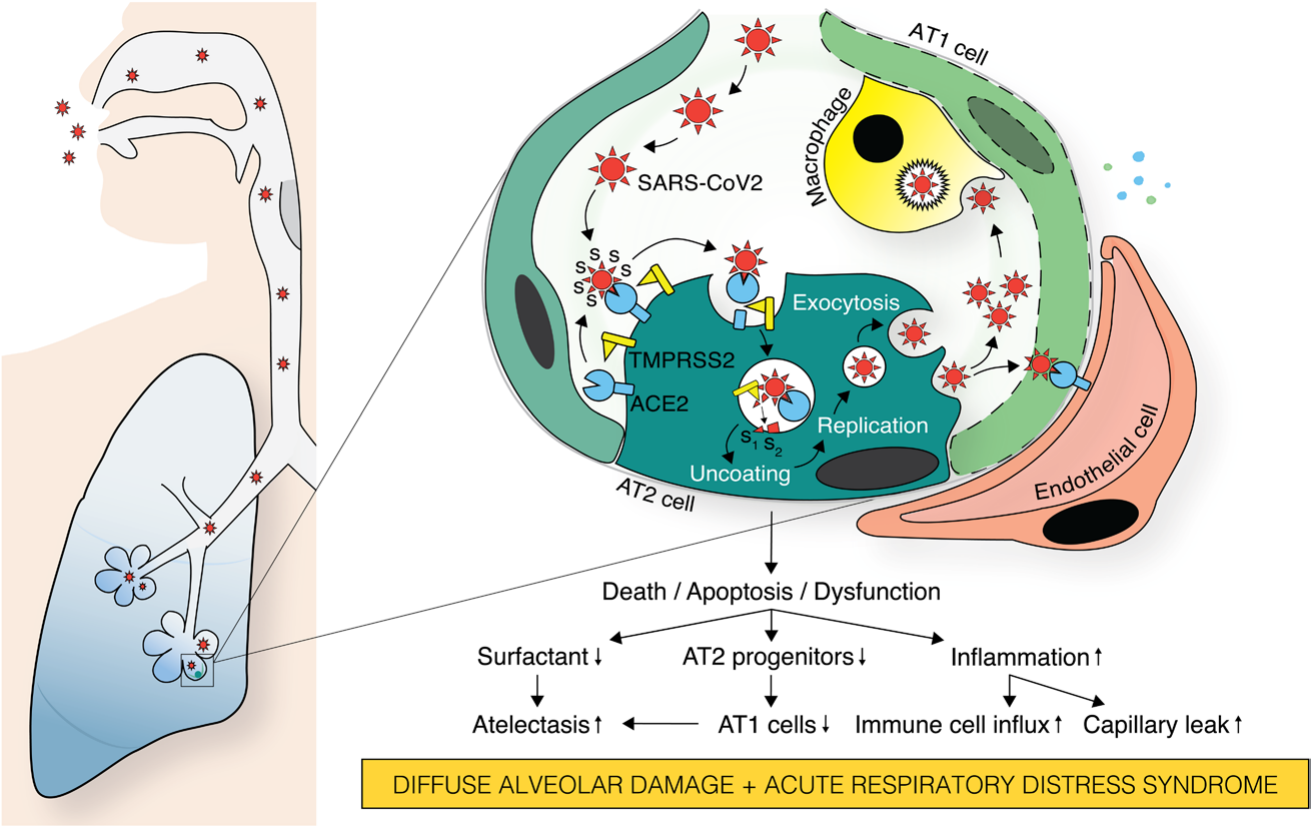
**Pathobiological consequences of alveolar epithelial injury by severe acute respiratory syndrome coronavirus-2 (SARS-CoV2) infection.** SARS-CoV2 host entry through alveolar epithelium critically depends on expression of ACE2 (angiotensin-converting enzyme-2) and TMPRSS2 (transmembrane serine protease 2). First, coronavirus binds through one of its 4 structural proteins, glycoprotein S (spike) to ACE2 on alveolar type II (AT2) cells, initiating fusion of virus, and host cell membranes. Second, TMPRSS2 simultaneously cleaves ACE2, promoting cell surface clearance of ACE2, and the viral glycoprotein S into subunits S_1_ and S_2_, resulting in viral uncoating and release of viral genome into the cytoplasm. The virus is then replicated using both viral and host cell machinery, translation of the viral core proteins S, M, N, and E in the endoplasmatic reticulum (ER), assembly of virus particles in the ER-Golgi-intermediate compartment, and packaging into small wallet vesicles routed to the plasma membrane for exocytosis. SARS-CoV2 infection-induced AT2 dysfunction or loss is deleterious to the injured lung for several reasons: (1) decrease in surfactant increases the risk for alveolar collapse and atelectasis. (2) Decrease in AT2 progenitor cells causes impaired alveolar type I (AT1) cell replacement, affecting alveolar repair and likely promote fibrosis. (3) ACE2 downregulation drives geographically restricted overactivity of the ACE/Angiotensin II/AT1 receptor axis, worsening the tissue destructive effect of the inflammatory response. (4) Viral-induced cytokine release by AT1/AT2 cells results in capillary leak and alveolar interstitial immune cell infiltration.

Although SARS-CoV-2 infection often begins in the upper airway epithelium, in a subset of patients, the virus infects or injures the alveolar epithelium diffusely, resulting in markedly impaired gas exchange and respiratory failure (Figure 1). As discussed above, infection is mediated by interaction of the viral S protein with ACE2, leading to internalization of the virion into endosomes. Host proteases (TMPRSS2 and possibly others, such as furin) cleave the S protein to create a fusion protein that enables the virus to enter the cytoplasm.^45,46^ Although alveolar type I and AT2 cells express ACE2, productive infection probably occurs mainly in surfactant-producing AT2 cells, as shown for SARS-CoV.^47^ There may be alternate cell-entry mechanisms, such as Fc-receptor-mediated internalization of antibody-bound virions.^48^ Infected cells produce virions, which infect adjacent epithelial cells, endothelial cells, and macrophages. Pathology studies of late-stage cases show viral protein and absence of prominent interstitial inflammation and vasculitis, suggesting that persistent infection of alveolar epithelium occurs in severe disease.

The likely fate of an infected alveolar epithelial cell is apoptosis, although the relative likelihoods of apoptosis, killing by effector T cells, other forms of cell death, or survival is unknown. In a hamster model of SARS-CoV-2 infection, there is widespread viral protein expression in the lung, with many cells undergoing apoptosis as assessed by TUNEL (terminal deoxynucleotidyl transferase dUTP nick-end labeling) assay.^49^ Viral proteins subvert cell functions, including apoptosis and interferon release, to increase virion production.^50^ Infected cells fuse to create syncytia, a process mediated by the fusion machinery mediating viral entry. Syncytium formation promotes cell-cell spread of the virus and evasion of immune surveillance. Infected cells detach, leaving behind a porous alveolar-capillary barrier. The alveolar epithelium provides most of the barrier function of the alveolar-capillary interface, so loss of epithelium is associated with plasma exudation or hemorrhage, and the formation of hyaline membranes containing fibrin, factor VIII, and cytokeratins.^51^ This process constitutes the pathological finding of DAD.^52,53^ However, Magro et al^27^ reported cases without DAD or pneumocyte involvement, characterized by microvascular thrombotic injury with complement deposition. More observations are needed to determine whether there is a distinct microvasculature phenotype without, or in addition to, prominent DAD.

Infection of epithelium has consequences other than virus production and barrier loss. Infected or injured (eg, by mechanical strain) epithelium produces cytokines. In vitro, alveolar epithelial cells infected with coronavirus or influenza virus secrete proinflammatory molecules (eg, IL-6, IL-8, IL-29, CCL5, CXCL9, CXCL10, and CXCL11).^54^ Loss of AT2 cells decreases surfactant secretion, contributing to alveolar collapse; it is not known whether infected but viable AT2 cells maintain surfactant secretion, but in vitro, influenza infection of AT2 cells results in reduced release of surfactant proteins A and D.^54^ Surfactant protein D, a lectin, binds SARS-CoV S protein; whether it binds SARS-CoV-2 S protein is unknown.^55^ Alveolar epithelium regulates coagulation and fibrinolysis on the alveolar surface, largely through production of urokinase and PAI1 (plasminogen activator inhibitor 1).^56^ SARS-CoV-2 pathology includes both hemorrhage and fibrin deposition in the alveolar space and microvasculature, implying perturbations in coagulation and fibrinolysis. A systems biology analysis of experimental SARS-CoV infection revealed that urokinase-related pathways predict lung injury.^57^ Thus, alveolar epithelium as well as endothelium may promote coagulation disorders in COVID-19.

Recovery from ARDS due to SARS-CoV-2 pneumonia requires reepithelialization and removal of hyaline membrane material and regression of stromal cell and leukocyte accumulations. Epithelium can be regenerated by residual AT2 cells, which proliferate and differentiate into alveolar type I cells. Alternatively—for example, if injury is so extensive that there are inadequate numbers of AT2 cells—repopulation is effected by specialized progenitor cells, at least in experimental models (eg, influenza infection). Alternative sources of regenerative cells in these studies include rare lineage-negative epithelial stem/progenitor cells, distal airway stem cells expressing p63 and keratin 5 (DASC^p63/Krt5^), and a Wnt-responsive subpopulation of AT2 cells.^58–60^ Building on these results, it may be possible to determine whether failure of regenerative programs, distinct from ongoing viral replication, microvascular thrombosis and endothelial dysfunction, and deleterious inflammation, is a cause of persistent lung dysfunction in COVID-19.

## Vascular Endothelium

**Figure 2.**
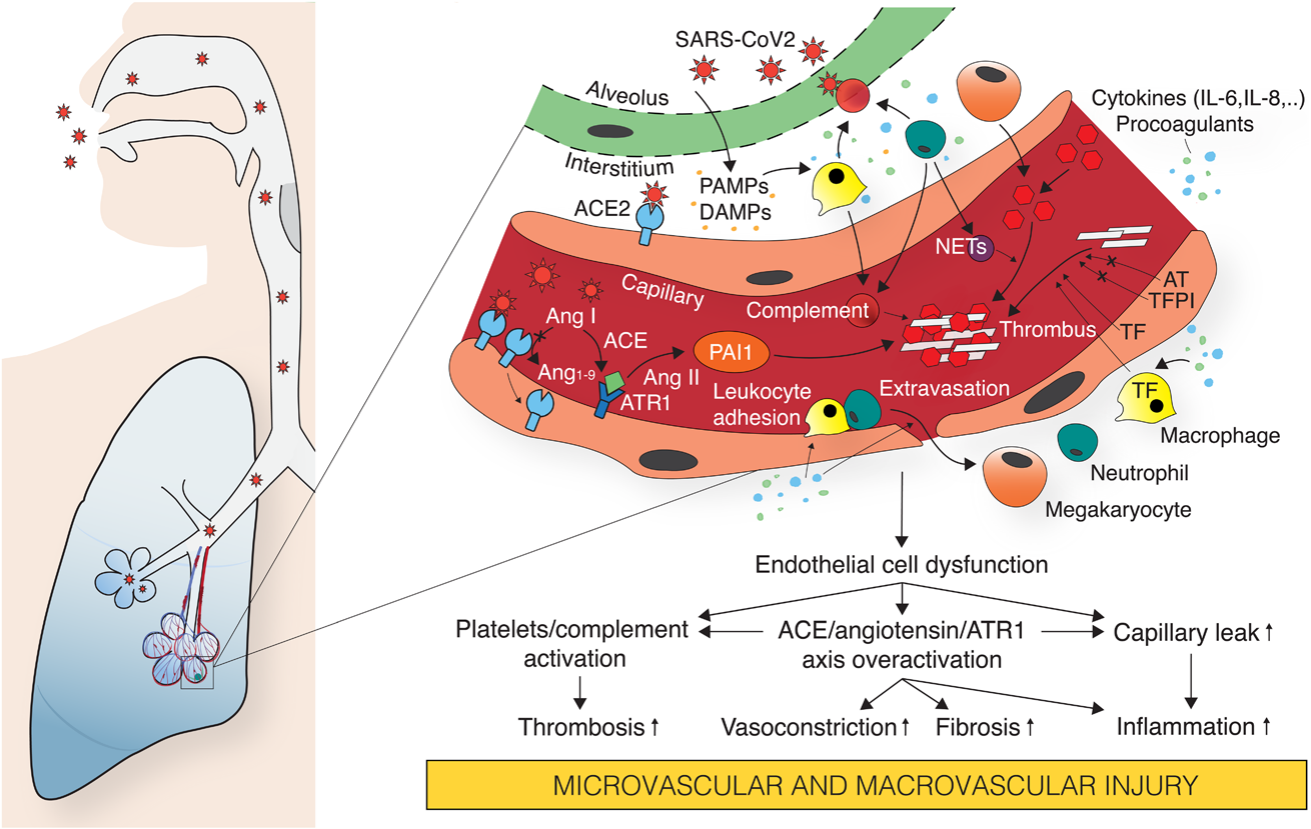
**Pathobiological consequences of vascular endothelial injury by severe acute respiratory syndrome coronavirus-2 (SARS-CoV2) infection.** SARS-CoV2 infection of endothelial cells, which might occur from luminal or alveolar interstitial side, triggers endothelial release of cytokines, which cause increased capillary permeability, thereby allowing adhesion and extravasation of neutrophils and monocytes into the alveolar interstitial space. Stimulated by PAMPs and DAMPs (pathogen-associated and damage-associated molecular patterns), neutrophils, and macrophages secret a multitude of cytokines, procoagulants, and complement, which promote viral attack and clearance but which induces further vascular injury enhancing the risk for thrombosis. Several factors might contribute to the prothrombotic environment, thereby promoting intravascular thrombus formation: (1) Neutrophil-mediated secretion of NETs (neutrophil extracellular traps) and complement enhances platelet aggregation. (2) Cytokine-triggered secretion of TF (tissue factor) by endothelial cells and macrophages stimulates the coagulation cascade and increases fibrin clot formation. (3) Endothelial damage decreases secretion of anticothrombotic mediators, such as AT (antithrombin) and TFPI (TF pathway inhibitor). (4) Lung residential megakaryocytes produce locally available platelets for aggregation. (5) Overactivation of the ACE (angiotensin-converting enzyme)/Ang II (angiotensin II)/AT_1_ receptor axis due to virus-induced ACE2 downregulation increases production of PAI1 (plasminogen activator inhibitor 1), reducing plasmin activation and fibrinolysis. AT indicates antithrombin; ATR, angiotensin receptor; and IL, interleukin.

The endothelium is an important target for SARS-CoV-2 infection, and vascular disorders are a major problem in COVID-19.^61^ The pulmonary vascular endothelium cooperates with vascular smooth muscle to effect hypoxic vasoconstriction via reversal of the nitric oxide pathway and other regulators, which matches perfusion to airspace ventilation; regulates clotting; regulates egress of leukocytes into the interstitium, a process that occurs mainly at the capillary level (rather than at the post-capillary level, as in most other tissues); and is part of the barrier for diffusion of water, solutes, and larger molecules between plasma and the interstitial space. Given these vital functions, endothelial dysfunction or loss may contribute to the lung pathophysiology of COVID-19 (Figure 2). Some histopathologic studies reveal direct viral infection of endothelial cells, with evidence of endothelial cell apoptosis, pyroptosis, and lymphocytic inflammation of the endothelium in the lungs and other organs.^62^ These histopathologic changes may be associated with organ ischemia, tissue edema, and a procoagulant state.^63^ Small series have identified pulmonary vascular perfusion abnormalities that may be related physiologically to the histopathology findings discussed above. For example, dual-energy CT scans of the lung (performed because of clinical suspicion for pulmonary thromboembolism) demonstrate abnormal radiographic appearance of small peripheral pulmonary arteries with vessel dilation and tortuosity among the prominent findings.^17^ These abnormalities are found near or within areas of lung parenchymal abnormalities, raising the possibility of abnormal pulmonary artery vasodilation in regions of hypoventilation, resulting in lost hypoxic vasoconstriction, unfavorable V/Q matching, and exaggerated hypoxemia. These observations, if confirmed, would unify observations of direct endothelial infection, endothelial inflammation or edema, and abnormal pulmonary vascular behavior, resulting in hypoxemia and elevated physiological dead space that appear to be out of proportion to radiographic evidence of diseased lung parenchyma and relatively normal lung compliance.^64^ It is possible that the observed degree of hypoxemia and preserved respiratory system compliance could be within the normal distribution for ARDS physiology.^65^

Notably, ACE2 has been seen by immunohistochemistry on pulmonary vascular endothelial cells, as well as vascular smooth muscle cells,^66^ raising the possibility that viral infection alters the cross-talk between these 2 cell types. This kind of change would likely result in abnormal vasoconstriction or vasodilation, causing gas exchange abnormalities. An exaggerated immune or inflammatory response may activate the complement cascade, further propagating endothelial damage as leukocytes are recruited and elaborate inflammatory cytokines and other signaling molecules.^27^ Although complement activation may be harmful in COVID-19, it is also critical in host response to infection^67^; its net effect in COVID-19 remains to be determined. In situ microthrombus formation instigated by local inflammation and endothelial damage would contribute to abnormal V/Q matching and gas exchange abnormalities. Future research could focus on providing evidence of abnormal pulmonary vascular blood flow and abnormal V/Q matching, correlating histopathologic findings with areas of gas exchange disorders and pulmonary vascular perfusion, and developing assays of disordered endothelial cell and vascular smooth muscle behavior associated with SARS-CoV-2 infection in vitro and in vivo.

## Venous Thromboembolic Disease and Pulmonary Microvascular Thrombosis

The incidence of venous thromboembolic disease in hospitalized patients with COVID-19 is high.^68^ For example, in a series of 198 hospitalized patients at one center, over a third of whom were in the intensive care unit (ICU), 20% developed deep vein thrombosis or pulmonary embolism despite thromboprophylaxis.^69^ Over half of these cases were symptomatic, and the cumulative incidence was higher among ICU patients (reaching 59% at 21 days of hospitalization). Similar results were found in a study of 184 ICU patients with COVID-19 pneumonia on thromboprophylaxis; 27% had pulmonary embolism or deep vein thrombosis.^70^ Laboratory testing of hospitalized patients with COVID-19 shows elevated fibrinogen, D-dimer, vWF (von Willebrand factor), and factor VIII. Platelet counts and aPTT (activated partial thromboplastin time) are typically normal.^71^ Disseminated intravascular coagulation and bleeding are rare.^72^ Thromboelastography reveals a hypercoagulable state with reduced reaction and clot formation times, increased maximum amplitude, and reduced clot lysis time in most patients tested.^73^

Pathology studies not only confirm the common occurrence of venous thromboembolic disease but also show microvascular thrombosis in the lungs and other organs. For example, in 12 consecutive autopsies, there were 4 cases of fatal pulmonary embolism and 3 additional cases of deep vein thrombosis; microvascular thrombi were regularly found in the lungs but rarely in other organs.^22^ In another series, no macroscopic pulmonary emboli were detected, but microscopic analysis revealed aggregates of CD4+ lymphocytes around small vessels with platelets and small thrombi in areas of DAD, microhemorrhage, and intravascular CD61+ megakaryocytes producing platelets.^74^ Intravascular and extravascular megakaryocytes are present in normal lungs and account for half of platelet production; more observations are needed to determine whether lung megakaryocyte function is altered in COVID-19.^75^ Alveolar-capillary microthrombosis was more prevalent in patients with COVID-19 than matched severity influenza lungs on autopsy.^23^ It is likely that common factors are involved in producing both large vessel thrombosis (deep vein thrombosis, pulmonary embolism, stroke, myocardial infarction) and microvascular thrombosis, but these 2 phenomena may also have distinct causes. Microvascular thrombosis in lung capillaries adjacent to ventilated alveoli would result in increased dead space, which is common in ARDS and is particularly prominent in COVID-19 pneumonia.^64^

It is not surprising that overwhelming viral infection results in increased clot formation. Patients hospitalized with COVID-19 are immobilized, have endothelial injury perhaps in part due to direct viral infection, and are hypercoagulable (see above), thus meeting all criteria of Virchow's triad. Viral PAMPs (pathogen-associated molecular pattern) trigger pathways involving pattern recognition receptors.^76^ DAMPs (damage-associated molecular patterns) and cytokines promote an increase in the number of circulating neutrophils and monocytes, which induces hypercoagulability.^77^ In a mouse model of venous thrombosis due to reduced flow, the coagulation system generates clot in cooperation with platelets, leukocyte-derived TF (tissue factor), and NETs (neutrophil extracellular traps).^78^ The apparent unusually high incidence of macroscopic and microvascular thrombosis may be due to a perfect storm of excessive immobility, endothelial damage from both direct viral infection and indirect effects of cytokines and leukocyte activation, and a markedly hypercoagulable state of blood due to intense stimulation of endothelium and mononuclear cells to increase release of factors, such as vWF, factor VIII, and TF.

The extent of macrovascular and microvascular thrombosis in COVID-19 is striking, suggesting that in addition to the factors noted above, there may be other SARS-CoV-2–specific mechanisms at play. Direct infection of endothelium might be such a factor.^62^ ACE2 is detected by immunohistochemistry on vascular endothelium^66^; however, single-cell sequencing does not confirm ACE2 or TMPRSS2 expression in endothelium.^79^ Ex vivo experiments on explants do not demonstrate endothelial infection.^80^ SARS-CoV-2 infection causes downregulation of ACE2, a negative regulator of angiotensin II. Angiotensin II upregulates platelet activator inhibitor 1 expression and increased angiotensin II causes microvascular thrombosis.^80–82^ There is evidence that NET formation is increased in patients with COVID-19, and several preclinical and approved drugs (such as colchicine) might reduce NET formation.^81,82^ NETs can increase coagulation in several ways, including thrombin generation and platelet adhesion.^76,82^ SARS-CoV-2 increases NET formation as evidenced by increased concentrations of myeloperoxidase-DNA and citrullinated histone H3.^82^ The prothrombotic state in inflammation can be worsened both by oxidative stress and hypoxia-inducible factors.^83,84^

Finally, complement activation may occur in some patients.^85,86^ In a mouse SARS-CoV infection model, complement activation occurs by day one, and mice deficient in C3 exhibit milder disease than controls.^86^ A complement-mediated microvascular injury was noted in a case series where deposition of C5b-9, C4d, and MASP2 (mannan-binding lectin serine protease 2) was seen in both skin and lung microvasculature.^27^ Some patients may develop a form of secondary hemophagocytic lymphohistiocytosis or macrophage activation syndrome^72^ in which thrombosis is triggered by TF through complex mechanisms that link innate immune cells and the coagulation cascade.^87^ TF is expressed in mononuclear cells and in vascular endothelial cells in response to damage or in response to inflammatory cytokines, such as IL (interleukin)-6 or IL-8, which are often elevated in response to COVID-19 infection.^76,88,89^ Future research could focus on finding which mechanisms of abnormal thrombosis predominate and whether these mechanisms are viable targets for prevention or therapy.

## Disorders of Control of Breathing and the Neuromuscular Breathing Apparatus

For many patients with COVID-19 with acute respiratory failure, invasive mechanical ventilation is required to prevent further deterioration in gas exchange, respiratory muscle fatigue, organ failure, and death.^9,90,91^ Prolonged periods of invasive mechanical ventilation (up to several weeks) have been reported.^90^ The effects of SARS-CoV-2 and prolonged mechanical ventilation on respiratory muscle structure and function are yet unknown but might be potentially detrimental and clinically important. The diaphragm is the main inspiratory muscle, and, compared with peripheral muscles, appears to be more affected by critical illness and mechanical ventilation.^92,93^ Diaphragm function may further deteriorate in ventilator-bound ICU patients.^92,94,95^ This is associated with difficult ventilator liberation, increased risks of ICU and hospital readmission, and increased risk for death.^93,95,96^

### Diaphragm Disuse Atrophy

Mechanical ventilation partially or completely unloads the respiratory muscles and silences the respiratory control centers in the brain stem (eg, due to sedation, neuromuscular blockade, or excessive ventilator assistance). Strong evidence suggests that diaphragm inactivity under mechanical ventilation is the critical contributor to diaphragm weakness.^92,97^ Diaphragm weakness has been demonstrated as reduced motion and thinning of the muscle as detected by ultrasound or as reduced capacity to generate pressures in response to a magnetic stimulus of the phrenic nerves.^95,98,99^ Clinical studies suggest that diaphragm weakness rapidly occurs during ICU stay (<4 days) and that the change in diaphragm thickness is correlated to the degree of diaphragm inactivity.^95^ The underlying cellular mechanisms, however, are poorly understood. Studies in animals and brain-dead organ donors who received controlled mechanical ventilation before organ harvest collectively demonstrate diaphragm muscle fiber atrophy and reduced contractile function.^100–103^ Diaphragm inactivity under mechanical ventilation is associated with excessive reactive oxygen species production, caspase-3 expression activating apoptosis, and upregulation of mRNAs coding for ligands related to the proteolytic ubiquitin-proteasome pathway.^100,101,103^ Translating these findings to critically ill patients is challenging, due to differences in underlying disease pathophysiology, background comorbid conditions, and treatments. In 2014, Hooijman et al^104^ were the first to study biochemical and functional changes in diaphragm biopsies obtained from invasively ventilated ICU patients. Both slow-twitch and fast-twitch muscle fiber atrophy was demonstrated, as well as activation of proteolytic pathways and an increased number of neutrophils and macrophages, supporting a role for inflammatory mediators in the development of diaphragm dysfunction.^104^ More recently, the same group revealed that mitochondrial alterations and oxidative stress did not play a causative role in the development of diaphragm atrophy and contractile dysfunction in these patients.^105^ It remains to be investigated whether SARS-CoV-2 patients present different cellular modifications and whether a direct attack of the virus on the muscle could be possible.

### Excessive Respiratory Drive; Potential Causes, and Effects on Lung and Diaphragm Function

Considering the effects discussed above, it is imperative to reduce sedation and to allow the patient to breathe spontaneously with the ventilator as soon as it is feasible and safe. Maintaining diaphragm activity under mechanical ventilation may be protective for preventing atrophy; however, this may also cause new problems as many patients develop strong breathing efforts as a result of excessive respiratory drive, which has been described in reports from patients with COVID-19.^106^ This may worsen lung injury and could potentially further impair diaphragm function (see below).^92,95,107,108^

Respiratory drive reflects the output from clusters of interneurons located in the brain stem that integrates continuous input from sources sensitive to chemical, mechanical, irritant, behavioral, and emotional stimuli.^109^ Potential mechanisms for excessive respiratory drive may include (but are not limited to) hypoxemia accompanied by an increased alveolar-to-arterial oxygen gradient (ie, ventilation-perfusion mismatch or intrapulmonary shunting), hypercapnia secondary to increased dead space fraction (eg, due to low tidal volume ventilation or hyperinflation), stimulation of lung and chest wall receptors, cortical stimuli, and brain stem inflammation.^64,65,110^ Regarding the latter, experimental studies suggest that viral invasion of the olfactory nerve progression into the brain stem respiratory centers could be a potential mechanism for central nervous system involvement in the manifestation of respiratory failure related to coronaviruses.^111^ Although evidence regarding SARS-CoV-2 is still scarce, coronavirus infections in the brain have been described before in both animal and human studies.^112^ In mice transgenic for human ACE2, SARS-CoV-1 enters the brain via the olfactory bulb, and infection results in rapid, transneuronal spread to connected regions. Only low doses of the virus intracranially resulted in lethal disease, whereas little infection was detected in the lungs, demonstrating that neurons are a highly susceptible target.^113^ A similar neuroinvasion pathway was described in few human brains with SARS-CoV-1 infection.^114^ Potentially, this viral neurotropism could partially explain the respiratory failure severity in the absence of dyspnea seen during the current pandemic, but autopsy studies are warranted to confirm this theory of pathogenesis and to target treatment protocols.^49,115^

Inadequate respiratory drive under mechanical ventilation may worsen lung and diaphragm function. In the absence of (severe) respiratory muscle weakness, excessive respiratory drive could lead to vigorous inspiratory efforts and high lung distending pressures. This is potentially harmful, especially in patients with already (severely) vulnerable lung tissue.^107,116^ First, large inspiratory efforts could cause regional alveolar overdistention and cyclic recruitment of collapsed lung areas, due to a heterogeneous and transient transmission of stress and strain.^108^ In addition, excessive respiratory drive may overwhelm lung-protective reflexes (Hering-Breuer inflation-inhibition reflex), leading to high tidal volumes and consequently lung injury and systemic inflammation.^117^ Moreover, excessive efforts could cause negative pressure pulmonary edema due to an increased transvascular pressure gradient and capillary leaks.^116^ The resulting deterioration in respiratory mechanics and gas exchange again increases respiratory drive, which may further worsen lung injury (so-called patient self-inflicted lung injury). In contrast, the effects of excessive respiratory drive on the diaphragm are less clear. Diaphragm injury has been demonstrated in nonventilated patients after excessive inspiratory loading, manifesting as a loss in force-generating capacity and sarcomere disruption on histology.^118^ In addition, animal research demonstrates that sepsis appears to sensitize the sarcolemma to load-induced injury.^119^ As strong breathing efforts are common in ventilated patients, similar cellular effects may develop but require further study.

Although data on the impact of COVID-19 ARDS on diaphragm function are lacking, prerequisites for development of diaphragm dysfunction (long-term mechanical ventilation, prolonged sedation, high inflammatory state) are present. Monitoring and targeting physiological levels of breathing effort is important for optimizing lung protection and potentially preventing diaphragm dysfunction, but this poses new challenges at the bedside and it remains uncertain whether such strategy can be implemented effectively.^120,121^ Frequent assessment of the patient’s ability to resume spontaneous breathing and to start ventilator liberation is of importance, not only to free up a ventilator for the next patient but also to limit the potential long-term and detrimental consequences of mechanical ventilation on the respiratory muscles, cognition, and functional status. Understanding these effects is an active area of research, and the identification of basic mechanisms of injury or patterns of clinical care that may be favorable or deleterious is crucial to lowering the risk of persistent disability among ICU survivors.

## Conclusions

COVID-19 affects all components of the respiratory system, including the neuromuscular breathing apparatus, the conducting airways, the respiratory airways and alveoli, the pulmonary vascular endothelium, and pulmonary blood flow. As of this writing, after fewer than 6 months of clinical experience and research into the disease, many publications have described gross and histological pathology, radiographic changes, and clinical manifestations of the disease. Few data, however, convincingly combine these observations into a more complete mechanistic model of the disease that permits researchers and clinicians to identify cause-and-effect relationships that can be targeted safely and effectively to improve clinical outcomes. Nevertheless, the accumulated data to this point identify several tantalizing avenues for investigation that may successfully lead to a fuller understanding of disease pathogenesis and identification of viable therapeutic targets. Although COVID-19 of the respiratory system appears to be a complex disease that may resist finding a single silver bullet intervention, these observations provide promising avenues to pursue. This review summarizes much of the known data on COVID-19–induced disorders of the respiratory system, offering researchers and clinicians an early and rough sketch of the challenges that confront us.

## Disclosures

None.
